# Complete genome sequence of *Arcanobacterium haemolyticum* type strain (11018^T^)

**DOI:** 10.4056/sigs.1123072

**Published:** 2010-09-28

**Authors:** Montri Yasawong, Hazuki Teshima, Alla Lapidus, Matt Nolan, Susan Lucas, Tijana Glavina Del Rio, Hope Tice, Jan-Fang Cheng, David Bruce, Chris Detter, Roxanne Tapia, Cliff Han, Lynne Goodwin, Sam Pitluck, Konstantinos Liolios, Natalia Ivanova, Konstantinos Mavromatis, Natalia Mikhailova, Amrita Pati, Amy Chen, Krishna Palaniappan, Miriam Land, Loren Hauser, Yun-Juan Chang, Cynthia D. Jeffries, Manfred Rohde, Johannes Sikorski, Rüdiger Pukall, Markus Göker, Tanja Woyke, James Bristow, Jonathan A. Eisen, Victor Markowitz, Philip Hugenholtz, Nikos C. Kyrpides, Hans-Peter Klenk

**Affiliations:** 1 HZI – Helmholtz Centre for Infection Research, Braunschweig, Germany; 2 DOE Joint Genome Institute, Walnut Creek, California, USA; 3 Los Alamos National Laboratory, Bioscience Division, Los Alamos, New Mexico, USA; 4 Biological Data Management and Technology Center, Lawrence Berkeley National Laboratory, Berkeley, California, USA; 5 Oak Ridge National Laboratory, Oak Ridge, Tennessee, USA; 6 DSMZ - German Collection of Microorganisms and Cell Cultures GmbH, Braunschweig, Germany; 7 University of California Davis Genome Center, Davis, California, USA

**Keywords:** obligate parasite, human pathogen, pharyngeal lesions, skin lesions, facultative anaerobe, *Actinomycetaceae*, *Actinobacteria*, GEBA

## Abstract

*Arcanobacterium haemolyticum* (ex MacLean *et al.* 1946) Collins *et al.* 1983 is the type species of the genus *Arcanobacterium*, which belongs to the family *Actinomycetaceae*. The strain is of interest because it is an obligate parasite of the pharynx of humans and farm animal; occasionally, it causes pharyngeal or skin lesions. It is a Gram-positive, nonmotile and non-sporulating bacterium. The strain described in this study was isolated from infections amongst American soldiers of certain islands of the North and West Pacific. This is the first completed sequence of a member of the genus *Arcanobacterium* and the ninth type strain genome from the family *Actinomycetaceae*. The 1,986,154 bp long genome with its 1,821 protein-coding and 64 RNA genes is a part of the *** G****enomic* *** E****ncyclopedia of* *** B****acteria and* *** A****rchaea * project.

## Introduction

Strain 11018^T^ (= DSM 20595 = CCM 5947 = ATCC 9345 = NBRC 15585) is the type strain of the species *A. haemolyticum*, which is the type species of its genus *Arcanobacterium* [1]. *Arcanobacterium* is one of six genera in the family *Actinomycetaceae* [2-4]. The genus currently consists of nine validly described species. The strain was first described in 1946 by MacLean as ‘*Corynebacterium haemolyticum*’ [5]. Based on chemical features and the presence of unique phenotypic characteristics, the strain was subsequently transferred to the new genus *Arcanobacterium* as *A. haemolyticum* [1] and emended by Lehnen *et al*. in 2006 [6]. The generic name drives from the Latin word ‘*arcanus*’, meaning ‘secretive’ and the Latin word ‘*bacterium*’, a small rod, meaning ‘secretive bacterium’ [1]. The species epithet is derived from the Latin word ‘*haema*’ meaning ‘blood’ and the Neo-Latin word ‘*lyticus*’ meaning ‘able to loose or able to dissolve’ referring to blood-dissolving or hemolytic when the cells grow on blood agar [1]. There are many medical case reports that *A. haemolyticum* is occasionally isolated in patients with brain abscess [7-9], cellulitis [10,11], endocarditis [12], meningitis [13], peritonitis [14], post-traumatic ankle joint infection [15], septic arthritis [16], septicemia [17], sinusitis [11], soft tissue infections [18], venous ulcer infection [19], vertebral osteomyelitis [20] and wound infection [21,22]. Only rarely are cases reported in animals, where pathogenicity of *A. haemolyticum* has not been well documented [23-25]. Here we present a summary classification and a set of features for *A. haemolyticum* strain 11018^T^, together with the description of the complete genomic sequencing and annotation.

## Classification and features

Strain 11018^T^ is an obligate parasite of the pharynx of human and farm animals; occasionally it causes pharyngeal or skin lesions [26]. The strain was isolated from infections in American soldiers [5]. The 16S rRNA gene sequence of strain 11018^T^ (AJ234059) is 99% identical to six culturable strains that were reported in GenBank (status July 2010). Five strains were isolated from infected horses [23]. Another culturable strain, Tr2-2X-1 (FJ477385), was isolated from gasoline contaminated soil. The 16S rRNA gene of strain 11018^T^ shares 93.3-97.9% sequence identity with the sequences of the type strains from the other members of the genus *Arcanobacterium* [27]. The next closest relative outside of the genus *Arcanobacterium* is *Dermacoccus barathri* MT2.1^T^ (92.3% sequence similarity) [27]. No phylotypes from environmental screening or metagenomic surveys could be linked to *A. haemolyticum* or even the genus *Arcanobacterium*, indicating a rare occurrence of these species in the habitats screened thus far (as of July 2010). A representative genomic 16S rRNA sequence of *A. haemolyticum* 11018^T^ was compared using BLAST with the most resent release of the Greengenes database [28] and the relative frequencies of taxa and keywords, weighted by BLAST scores, were determined. The five most frequent genera were *Arcanobacterium* (42.4%), *Dermacoccus* (12.6%), *Actinomyces* (10.8%), *Terrabacter* (9.9%) and *Sanguibacter* (5.7%). The five most frequent keywords within the labels of environmental samples were 'skin' (6.6%), 'human' (5.0%), 'feedlot' (4.6%), 'elbow' (3.4%) and 'microbiota' (3.3%). The BLAST keywords analysis supports the biological insights into *A. haemolyticum* strain 11018^T^ as described above.

**Figure 1:**
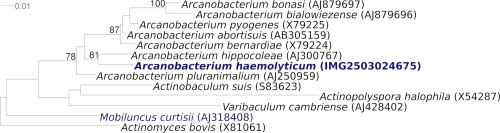
Phylogenetic tree highlighting the position of *A. haemolyticum* strain 11018^T^ relative to the type strains of the other species within the genus *Arcanobacterium* and to the type strains of the other genera within the family *Actinomycetaceae*. The trees were inferred from 1,388 aligned characters [29,30] of the 16S rRNA gene sequence under the maximum likelihood criterion [31] and rooted in accordance with the current taxonomy. The branches are scaled in terms of the expected number of substitutions per site. Numbers above branches are support values from 1,000 bootstrap replicates [32] if larger than 60%. Lineages with type strain genome sequencing projects registered in GOLD [33] are shown in blue, published genomes in bold.

Figure 1 shows the phylogenetic neighborhood of *A. haemolyticum* strain 11018^T^ in a 16S rRNA based tree. The sequences of the four 16S rRNA gene copies in the genome differ from each other by up to two nucleotides, and differ by up to five nucleotides from the previously published sequence generated from CIP 103370 (AJ234059) which contains one ambiguous base call.

**Figure 2:**
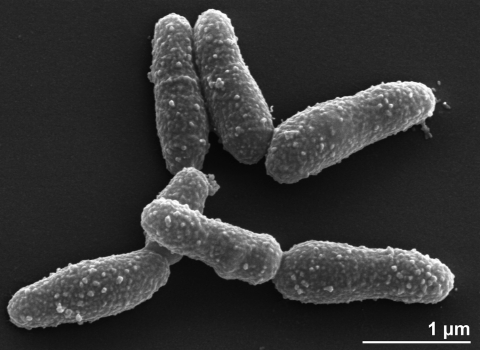
Scanning electron micrograph of *A. haemolyticum* strain 11018^T^

The cells of strain 11018^T^ are slender or irregular rods (0.3-0.8 × 1.0-5.0 µm) [Table 1 and Figure 2]. The cells are Gram-positive, nonmotile, not acid-fast and without endospores [1]. In young cultures, cells may show clubbed ends sometimes arranged in V formation, but there are no filaments. In older cultures, cells segment into short, irregular rods and cocci [1]. Strain 11018^T^ is facultatively anaerobic. The cells grow slowly on nutrient agar, but grow better on horse blood agar, giving small, convex, translucent colonies surrounded by a zone of complete hemolysis after two days at 37°C [1]. The selective medium for this strain was developed by Coman [39] and contains 5% sheep blood and 3.5% of NaCl. Cell growth is enhanced by the addition of CO_2_ [1]. The optimum growth temperature is 37°C [1,26]. Cells do not withstand heating at 60°C for 15 min [1,5]. Strain 11018^T^ is chemoorganotrophic and requires nutritionally rich media for growth [1,26]. The fermentative metabolism of this strain produces acid but does not produce gas from glucose and several other carbohydrates on which growth occurs [1,26]. Acid production is mainly acetic, lactic and succinic acids [1,26]. Catalase, nitrate reduction and gelatine hydrolysis reactions are negative [6]. Strain 11018^T^ produces *N*-acetyl-*β*-galactosidase, alkaline phosphatase, extracellular DNase, *β*-galactosidase, *α*-glucosidase and pyrazinamidase. It does not produce acid phosphatase, *α*-chymotrypsin, cystine arylamidase, esterase (C4), esterase lipase (C8), *α*-fucosidase, *α*-galactosidase, *β*-glucosidase, *β*-glucuronidase, leucine arylamidase, lipase (C14), *α*-mannosidase, naphthol-AS-BI-phosphohydrolase, trypsin, valine arylamidase and urease [1,6]. Strain 11018^T^ is not able to ferment adonitol, L-arabitol, erythritol, D-fructose, glycerol, glycogen, D-mannitol and D-xylose. It is resistant to oxytetracycline (30µg per disc) but susceptible to nalidixic acid (30µg per disc), sulfamethoxazole trimethoprim (25µg per disc), amikacin (10µg per disc) or cefoxitin (30µg per disc) [1,42].

**Table 1:** Classification and general features of *A. haemolyticum* strain 11018^T^ according to the MIGS recommendations [34].

**MIGS ID**	**Property**	**Term**	**Evidence code**
	Current classification	Domain *Bacteria*	TAS [35]
		Phylum *Actinobacteria*	TAS [36]
		Class *Actinobacteria*	TAS [3]
		Subclass *Actinobacteridae*	TAS [3,4]
		Order *Actinomycetales*	TAS [2-5,37]
		Suborder *Actinomycineae*	TAS [3,4]
		Family *Actinomycetaceae*	TAS [2-5,37]
		Genus *Arcanobacterium*	TAS [1,6,38]
		Species *Arcanobacterium haemolyticum*	TAS [1,5,38]
		Type strain 11018	TAS [1]
	Gram stain	positive	TAS [1]
	Cell shape	slender, irregular rods (0.3-0.8 ×1.0-5.0 µm)	TAS [1]
	Motility	none	TAS [1]
	Sporulation	none	TAS [1]
	Temperature range	not reported	
	Optimum temperature	37°C	TAS [1]
	Salinity	3.5%	TAS [39]
MIGS-22	Oxygen requirement	facultatively anaerobic	TAS [1]
	Carbon source	carbohydrates	TAS [1,5,6]
	Energy source	chemoorganotroph	TAS [26]
MIGS-6	Habitat	pharynx of humans and farm animals	TAS [26]
MIGS-15	Biotic relationship	obligate parasite	TAS [26]
MIGS-14	Pathogenicity	pharyngeal or skin lesions	TAS [26]
	Biosafety level	2	TAS [40]
	Isolation	infections amongst American soldiers	TAS [5]
MIGS-4	Geographic location	North and West Pacific	TAS [5]
MIGS-5	Sample collection time	1946 or before	TAS [1,5]
MIGS-4.1	Latitude	not reported	
MIGS-4.2	Longitude	not reported	
MIGS-4.3	Depth	not reported	
MIGS-4.4	Altitude	not reported	

Evidence codes - IDA: Inferred from Direct Assay (first time in publication); TAS: Traceable Author Statement (i.e., a direct report exists in the literature); NAS: Non-traceable Author Statement (i.e., not directly observed for the living, isolated sample, but based on a generally accepted property for the species, or anecdotal evidence). These evidence codes are from of the Gene Ontology project [41]. If the evidence code is IDA, then the property was directly observed by one of the authors or an expert mentioned in the acknowledgements

### Chemotaxonomy

Strain 11018^T^ possesses peptidoglycan type A5α based on L-Lys-L-Lys-D-Glu (unpublished, Norbert Weiss [43]). The predominant menaquinone is MK-9(H_4_) (85%) complemented by 15% MK-8(H_4_) [6]. The major cellular fatty acids when grown on blood agar at 35°C are straight-chain unsaturated acids C_18:1_ *ω*9*c* (37.0%), and saturated acids C_18:0_ (24.7%), C_16:0_ (22.5%) [6], which is similar to the cellular fatty acids spectrum reported from cells grown on sheep blood agar [31]: C_18:1_ *cis*9 (29%), C_16:0_ (23%), C_18:2_ (18%), C_18:0_ (17%), C_10:0_ (3%) and C_14:0_ (2%).

## Genome sequencing and annotation

### Genome project history

This organism was selected for sequencing on the basis of its phylogenetic position [44], and is part of the *** G****enomic* *** E****ncyclopedia of* *** B****acteria and* *** A****rchaea * project [45]. The genome project is deposited in the Genome OnLine Database [33] and the complete genome sequence is deposited in GenBank. Sequencing, finishing and annotation were performed by the DOE Joint Genome Institute (JGI). A summary of the project information is shown in Table 2.

**Table 2:** Genome sequencing project information

**MIGS ID**	**Property**	**Term**
MIGS-31	Finishing quality	Finished
MIGS-28	Libraries used	Three genomic libraries: 454 pyrosequence standard, PE (12.5 kb insert size) libraries and one Illumina standard library
MIGS-29	Sequencing platforms	454 GS FLX Titanium, Illumina GAii
MIGS-31.2	Sequencing coverage	83.8 × pyrosequence, 36.8 x Illumina
MIGS-30	Assemblers	Newbler version 2.0.0-PostRelease- 11/04/2008, phrap, Velvet
MIGS-32	Gene calling method	Prodigal 1.4, GenePRIMP
	INSDC ID	CP002045
	Genbank Date of Release	June 4, 2010
	GOLD ID	Gc01291
	NCBI project ID	37925
	Database: IMG-GEBA	646564505
MIGS-13	Source material identifier	DSM 20595
	Project relevance	Tree of Life, GEBA

### Growth conditions and DNA isolation

*A. haemolyticum* strain 11018^T^, DSM 20595, was grown anaerobically in DSMZ medium 104 (PYG modified medium) [46] at 37°C. DNA was isolated from 1-1.5 g of cell paste using MasterPure Gram Positive DNA Purification Kit (Epicentre MGP04100), with a modified protocol for cell lysis, st/LALM, as described in Wu *et al.* [45].

### Genome sequencing and assembly

The genome was sequenced using a combination of Illumina and 454 sequencing platforms. All general aspects of library construction and sequencing can be found at the JGI website (http://www.jgi.doe.gov/). Pyrosequencing reads were assembled using the Newbler assembler version 2.0.0-PostRelease-11/04/2008 (Roche). The initial Newbler assembly consisted of 116 contigs in 28 scaffolds and was converted into a phrap assembly by making fake reads from the consensus, collecting the read pairs in the 454 paired end library. Illumina GAii sequencing data was assembled with Velvet [47] and the consensus sequences were shredded into 1.5 kb overlapped fake reads and assembled together with the 454 data. Draft assemblies were based on 166.4 Mb 454 draft and all of the 454 paired end data. Newbler parameters are -consed -a 50 -l 350 -g -m -ml 20.

The Phred/Phrap/Consed software package (www.phrap.com) was used for sequence assembly and quality assessment in the following finishing process. After the shotgun stage, reads were assembled with parallel phrap (High Performance Software, LLC). Possible mis-assemblies were corrected with gapResolution (http://www.jgi.doe.gov/), Dupfinisher [48], or sequencing cloned bridging PCR fragments with subcloning or transposon bombing (Epicentre Biotechnologies, Madison, WI) [49]. Gaps between contigs were closed by editing in Consed, by PCR and by Bubble PCR primer walks (J.-F. Chang, unpublished). A total of 140 additional reactions were necessary to close gaps and to raise the quality of the finished sequence. Illumina reads were also used to improve the final consensus quality using an in-house developed tool - the Polisher [50]. The error rate of the completed genome sequence is less than 1 in 100,000. Together, the combination of the Illumina and 454 sequencing platforms provided 120.6 ×coverage of the genome. The final assembly contains 2.03 million Illumina reads and 0.52 million pyrosequencing reads.

### Genome annotation

Genes were identified using Prodigal [51] as part of the Oak Ridge National Laboratory genome annotation pipeline, followed by a round of manual curation using the JGI GenePRIMP pipeline [52]. The predicted CDSs were translated and used to search the National Center for Biotechnology Information (NCBI) nonredundant database, UniProt, TIGRFam, Pfam, PRIAM, KEGG, COG, and InterPro databases. Additional gene prediction analysis and functional annotation was performed within the Integrated Microbial Genomes - Expert Review (IMG-ER) platform [53].

## Genome properties

**Figure 3:**
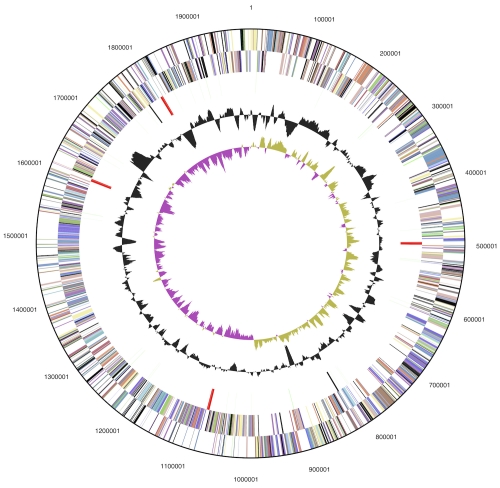
Graphical circular map of the genome. From outside to the center: Genes on forward strand (color by COG categories), Genes on reverse strand (color by COG categories), RNA genes (tRNAs green, rRNAs red, other RNAs black), GC content, GC skew.

The genome consists of a 1,986,154 bp long chromosome with a 53.1% GC content (Table 3 and Figure 3). Of the 1,885 genes predicted, 1,821 were protein-coding genes, and 64 RNAs; 90 pseudogenes were also identified. The majority of the protein-coding genes (68.5%) were assigned with a putative function while the remaining ones were annotated as hypothetical proteins. The distribution of genes into COGs functional categories is presented in Table 4.

**Table 3:** Genome Statistics

**Attribute**	**Value**	**% of Total**
Genome size (bp)	1,986,154	100.00%
DNA coding region (bp)	1,744,192	87.82%
DNA G+C content (bp)	1,055,308	53.13%
Number of replicons	1	
Extrachromosomal elements	0	
Total genes	1,885	100.00%
RNA genes	64	3.40%
rRNA operons	4	
Protein-coding genes	1,821	96.60%
Pseudo genes	90	4.77%
Genes with function prediction	1,292	68.54%
Genes in paralog clusters	154	8.17%
Genes assigned to COGs	1,308	69.39%
Genes assigned Pfam domains	1,402	74.38%
Genes with signal peptides	391	20.74%
Genes with transmembrane helices	492	26.10%
CRISPR repeats	1	

**Table 4:** Number of genes associated with the general COG functional categories

**Code**	**Value**	**%age**	**Description**
J	136	9.7	Translation, ribosomal structure and biogenesis
A	1	0.1	RNA processing and modification
K	99	7.1	Transcription
L	119	8.5	Replication, recombination and repair
B	0	0.0	Chromatin structure and dynamics
D	21	1.5	Cell cycle control, cell division, chromosome partitioning
Y	0	0.0	Nuclear structure
V	36	2.6	Defense mechanisms
T	51	3.6	Signal transduction mechanisms
M	75	5.4	Cell wall/membrane/envelope biogenesis
N	0	0.0	Cell motility
Z	0	0.0	Cytoskeleton
W	0	0.0	Extracellular structures
U	27	1.9	Intracellular trafficking and secretion, and vesicular transport
O	56	4.0	Posttranslational modification, protein turnover, chaperones
C	86	6.1	Energy production and conversion
G	125	8.9	Carbohydrate transport and metabolism
E	77	5.5	Amino acid transport and metabolism
F	58	4.1	Nucleotide transport and metabolism
H	56	4.0	Coenzyme transport and metabolism
I	34	2.4	Lipid transport and metabolism
P	93	6.6	Inorganic ion transport and metabolism
Q	12	0.9	Secondary metabolites biosynthesis, transport and catabolism
R	152	10.9	General function prediction only
S	87	6.2	Function unknown
-	577	30.6	Not in COGs
